# Colorimetric detection of oral bacteria using functionalized gold nanoparticles as a plasmonic biosensor array†

**DOI:** 10.1039/d3na00477e

**Published:** 2024-02-08

**Authors:** Christina Wenck, Dorthe Leopoldt, Mosaieb Habib, Jan Hegermann, Meike Stiesch, Katharina Doll-Nikutta, Alexander Heisterkamp, Maria Leilani Torres-Mapa

**Affiliations:** a Institute of Quantum Optics, Leibniz University Hannover Germany torres@iqo.uni-hannover.de; b Institute of Inorganic Chemistry, Leibniz University Hannover Germany; c Department of Prosthetic Dentistry and Biomedical Materials Science, Hannover Medical School Germany; d Research Core Unit Electron Microscopy, Institute of Functional and Applied Anatomy, Hannover Medical School Germany; e Lower Saxony Centre for Biomedical Engineering, Implant Research and Development (NIFE) Germany

## Abstract

Early detection of specific oral bacterial species would enable timely treatment and prevention of certain oral diseases. In this work, we investigated the sensitivity and specificity of functionalized gold nanoparticles for plasmonic sensing of oral bacteria. This approach is based on the aggregation of positively charged gold nanoparticles on the negatively charged bacteria surface and the corresponding localized surface plasmon resonance (LSPR) shift. Gold nanoparticles were synthesized in different sizes, shapes and functionalization. A biosensor array was developed consisting of spherical- and anisotropic-shaped (1-hexadecyl) trimethylammonium bromide (CTAB) and spherical mercaptoethylamine (MEA) gold nanoparticles. It was used to detect four oral bacterial species (*Aggregatibacter actinomycetemcomitans*, *Actinomyces naeslundii*, *Porphyromonas gingivalis* and *Streptococcus oralis*). The plasmonic response was measured and analysed using *RGB* and UV-vis absorbance values. Both methods successfully detected the individual bacterial species based on their unique responses to the biosensor array. We present an in-depth study relating the bacteria zeta potential and AuNP aggregation to plasmonic response. The sensitivity depends on multiple parameters, such as bacterial species and concentration as well as gold nanoparticle shape, concentration and functionalization.

## Abstract 2

A plasmonic biosensor array consisting of functionalized gold nanoparticles with strong positive surface charge can detect dysbiosis-relevant oral bacterial species using colorimetric analysis.

## Graphical abstract

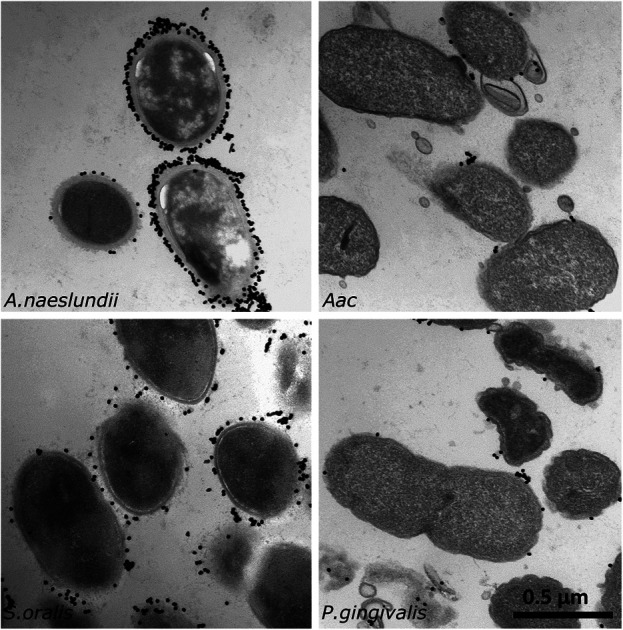

## 1. Introduction

The oral microbiome is composed of several hundreds of bacterial species forming a diverse, microbial community.^1^ Oral microbes form complex, surface-attached agglomerates, called biofilms that initially support a homeostatic equilibrium maintaining a healthy host tissue. An imbalance, termed as dysbiosis, may result to oral pathological conditions such as chronic periodontitis and periimplantitis.^2–5^ Certain pathogens characterize the dysbiotic state and push the host's inflammation response. These keystone pathogens are also implicated to other diseases such as Alzheimer's disease,^6^ colorectal cancer proliferation^7^ and autoimmune disorders.^8^ Particularly, *Porphyromonas gingivalis* (*P. gingivalis*) is considered a critical pathogen that becomes abundant upon bacterial dysbiosis.^9^ This bacterium expresses gingipains – cysteine proteases suggested to play a major role in the its pathogenicity and to contribute to its virulence.^10,11^

Other bacterial species have been also shown to support dysbiosis state. For example, *Aggregatibacter actinomycetemcomitans* (*Aac*) contribute virulence factors such as leukotoxin and lipopolysaccharide inducing proinflammatory mediators.^12^*Aac* is considered one of the main causes of endocarditis, soft tissue infections, abscess formation and periodontitis.^13^ Both *P. gingivalis* and *Aac* are found in a healthy oral cavity, but increases in number in cases of periodontal disease.^14^ In contrast, *Actinomyces naeslundii* (*A. naeslundii*) and *Streptococcus oralis* (*S. oralis*), are involved in maintaining a healthy oral cavity and respiratory tract.^15–17^ In oral biofilms, both bacterial species are considered as early colonizers.^15^*S. oralis* is also often detected in dental plaques^18^ and can cause disease in the blood. In cases of disruption of tissue barriers, pure *A. naeslundii* could cause infections of organs and bloodstream but often has a lower pathogenicity, hence it commonly occurs as a part of mixed infections.^17^

Not only is dysbiosis attributed to the presence of pathogenic bacteria but also to an initial proliferation of healthy bacteria followed by the biofilm composition shift. Hence, detecting dysbiosis by monitoring the microbiome in the oral cavity would not only require the detection of a single bacterial species but also the measurement of the relative proportion of several key bacteria. At present, there is still a need to develop new methods that could monitor and detect the presence of multiple bacterial species and provide their relative increase over time for health monitoring and disease prevention. For future point-of-care detection, a fast, inexpensive read-out without the need for complicated equipment would be ideal.

Conventional methods for bacteria detection, like colony counting, fluorescence microscopy, PCR-based and immunological assays can be very specific and accurate but are considered time-consuming (>24 h) approaches, require specialized technical equipment and extensive sample preparation.^19^ Particularly for oral bacteria detection, loop mediated isothermal amplification has been implemented to detect *Streptococcus mutans* gene^20^ and *P. gingivalis* fibril proteins.^21^ A CRISPR-cas-based assay was also used to identify seven oral bacterial species in unprocessed saliva samples.^22^ Recently, a microfluidic based platform based on PCR detection was implemented to detect both periodontitis and caries-associated bacteria.^23^

Gold nanoparticles (AuNPs) have attracted attention in the sensing community because of their unique optical properties. Particularly, AuNPs exhibit localized surface plasmon resonance (LSPR) which depends on their shape, size and refractive index of the local environment. Especially for colorimetric detection, the LSPR shift which is measured as colour change in the samples corresponding to UV-vis absorbance shifts is affected by the AuNPs aggregation state. Therefore, aggregation of smaller AuNPs around micron-sized bacteria can lead to a visual colour change which can be observed by the naked eye and quantitatively measured using a camera. Several techniques have been implemented to detect bacteria using AuNPs. For example, Wang *et al.*^24^ and Khan *et al.*^25^ used antibody conjugated AuNPs to detect *Salmonella typhimurium*, based on antibody-antigen recognition leading to a conjugation of the AuNPs to the bacteria surface and therefore to a LSPR shift. Specificity was demonstrated by adding *E. coli* to the AuNPs which did not lead to a LSPR shift. Another technique for bacteria detection was demonstrated by Wu *et al.* using aptamer conjugated AuNPs to detect *E. coli* and *S. typhimurium*.^26^ Here, the aptamers act as an electrostatic stabilization agent. By adding the corresponding bacterial species, the aptamer conformation changes which leads to a separation of aptamers and AuNPs. With the addition of salt, AuNPs aggregate in solution causing a LSPR shift. The specificity was demonstrated on various bacterial species (*e.g. Shigella flexneri*, *Salmonella paratyphi* A, and *Straphylococcus aureus*) using two types of aptamer-conjugated AuNPs for detection. Other bacterial species did not induce the separation of aptamers and AuNPs effectively. Although techniques based on antibody and aptamer conjugated AuNPs are rapid and highly specific, they can only detect a single bacterial species at a time, which for the oral microbiome is highly limiting and not applicable due to the multitudes of bacteria present. On the other hand, Verma *et al.* demonstrated a rapid method which uses the electrostatic interaction of the negatively charged bacterial cell wall and the positively charged CTAB functionalized AuNPs to detect multiple bacterial species simultaneously.^27–29^ This method is more cost-efficient^19^ since the molecules used for functionalization are cheaper and less time-intensive to produce than specific antibodies and aptamers. The aggregation of positively charged AuNPs around the bacteria would depend on the bacterial cell wall properties and induce a unique set of plasmonic responses. Development of a sensor array composed of functionalized AuNPs with varying plasmonic properties would further increase the method's specificity in identifying bacterial species.^29–31^

In this work, we develop an AuNP biosensor array consisting of CTAB and MEA functionalized AuNPs to identify the colour shift responses of four dysbiosis-relevant oral bacterial species: Gram-positive bacteria, *A. naeslundii* and *S. oralis* and Gram-negative bacteria, *Aac* and *P. gingivalis*. The rod-shaped *A. naeslundii* is 0.4 to 1 μm in size. *S. oralis* is a 0.75 μm spherical, anaerobic bacterium.^16^*Aac* is a coccoid to rod-shaped bacterium with a typical size between 0.1 to 1.0 μm and grows as facultative anaerobic, non-motile and non-spore-forming.^13^*P. gingivalis* is an anaerobic, rod-shaped and non-motile bacterium up to 1 μm in size.^14,32,33^ We characterize at depth the plasmonic responses of these four bacteria to our synthesized AuNP array. Furthermore, we explore a range of parameters such as bacteria concentration and AuNP dilution to identify conditions with enhanced sensitivity. We present detailed analysis of the plasmonic responses based on colorimetric approach supported by UV-vis spectrometry. Overall, our work contributes to functionalized AuNP-based colorimetric sensing studies by extending its application to oral bacterial species.

## 2. Materials and methods

Gold(iii) chloride hydrate (99.995%), cysteamine (∼95%), l-ascorbic acid (reagent grade), silver nitrate (99.9999%), sodium borohydrate (99%) and trisodium citrate dihydrate were purchased from Sigma Aldrich. (1-Hexadecyl)trimethylammonium bromide (98%) were purchased from Alfa Aesar.

To characterize the synthesized AuNPs, dynamic light scattering (Zetasizer Nano ZSP, Malvern Panalytical) was used to measure the zeta potential and hydrodynamic radius. UV-vis spectra were measured using a spectrophotometer (Biowave II, WPA) and a fluorometer plate reader (Infinite M200 PRO, Tecan). Images of the well plates were taken using a commercially available CCD camera (Lumix DC-TZ91, Panasonic).

### 2.1 Gold nanoparticle synthesis

Synthesis of CTAB-AuNPs. A seeded-growth synthesis was used to produce CTAB-AuNPs. Here, the synthesis protocols of Verma *et al.*^27–29^ were adjusted and used.

The synthesis of the seed was performed by first adding 436 μL of 11 mM gold(iii) chloride hydrate solution to 19.084 mL MilliQ water and stirred for 1 min. Then, 480 μL of 10 mM trisodium citrate dihydrate solution was added and the solution was stirred for another 3 min. Afterwards, 60 μL of 0.1 M freshly prepared and ice-cold sodium borohydride solution was quickly added under vigorous stirring with further stirring for 5 min. After overnight incubation in the dark under ambient conditions, the colour of the seed turned from brown to red. After the incubation time, the seed was filtered (0.2 μm pore size).

The synthesis of the spherical and anisotropic CTAB-AuNPs only differs in the concentrations and volumes of some reagents. First, 210 mL of 1.46 mM (spherical) or 7.33 mM (anisotropic) CTAB solution was prepared. Under moderate stirring, 8.97 mL of 11 mM gold(iii) chloride hydrate solution and 1.34 mL (spherical) or 0.67 mL (anisotropic) of 10 mM silver nitrate solution were added. Then, 1.44 mL of 100 mM l-ascorbic acid solution were added dropwise. After the solution turns turbid white (spherical) or clear (anisotropic), 5.6 mL (spherical) or 2.24 mL (anisotropic) of seed were immediately added. The solution was stirred for another 1.5 min and then allowed to sit under ambient condition for 10 min. Then, the colloidal suspension was centrifuged at 12 500 RCF for 30 min. Finally, the supernatant was removed and the AuNPs were redispersed in MilliQ water.

#### 2.2.1 Synthesis of spherical MEA-AuNPs

For the synthesis of spherical MEA-AuNPs, the protocols of Sun *et al.*^34^ and Niidome *et al.*^35^ were adjusted and used.

The MEA-AuNPs were synthesized by first preparing 40 mL of 1.42 mM gold(iii) chloride hydrate. To this, 400 μL of 213 mM MEA (final concentration of 2.11 mM) were added and the solution was stirred for 20 min in the dark at room temperature. Then, 10 μL of 10 mM freshly prepared and ice-cold sodium borohydrate were added under vigorous stirring. This was followed by another 10 min of vigorous stirring, then 30 min of mild stirring and finally incubation for at least 1.5 h in the dark at room temperature.

### 2.2 Bacteria strains and culture conditions

*Streptococcus oralis* ATCC® 9811 was obtained from the American Type Culture Collection (ATCC®, Manassas, VA, USA). *Actinomyces naeslundii* DSM 43013 and *Porphyromonas gingivalis* DSM 20709 were obtained from the German Collection of Microorganisms and Cell Cultures GmbH (Braunschweig, Germany). *Aggregatibacter actinomycetemcomitans* MCCM 2474 strain was obtained from the Microbial Culture Collection Marburg.

All bacterial strains were routinely stored as glycerol stocks at −80 °C. As culture medium Todd-Hewitt Broth (Oxoid Limited, Hampshire, UK) supplemented with 10% yeast extract (THBy, Carl Roth GmbH + Co. KG, Karlsruhe, Germany), Brain–Heart Infusion (BHI, Oxoid Limited) supplemented with 10 μg mL^−1^ vitamin K (Oxoid Limited) and Fastidious Anaerobe Broth (FAB, Oxoid Limited) were used as specified in Table 1. For the bacteria culture, 20 μL (*S. oralis*, *A. naeslundii*, *Aac*) or 30 μl (*P. gingivalis*) of the glycerol stocks were added into 10 mL of the corresponding medium in a 50 mL centrifugal tube and incubated under the conditions shown in Table 1. Then, bacterial cells were washed three times with autoclaved deionized water by centrifuging at 2000 RCF for 5 min.

**Table 1:** Bacteria culture conditions

Bacteria	Medium	Incubation time	Conditions
*Aac*	THB + 10% yeast extract	48 h	37 °C, 5% CO_2_
*A. naeslundii*	BHI + 10 μg mL^−1^ vitamin K	24 h	37 °C, anaerobe
*P. gingivalis*	FAB	96 h	37 °C, anaerobe
*S. oralis*	BHI + 10 μg mL^−1^ vitamin K	24 h	37 °C, anaerobe

### 2.3 Bacteria sensing experiments

**Fig. 1:**
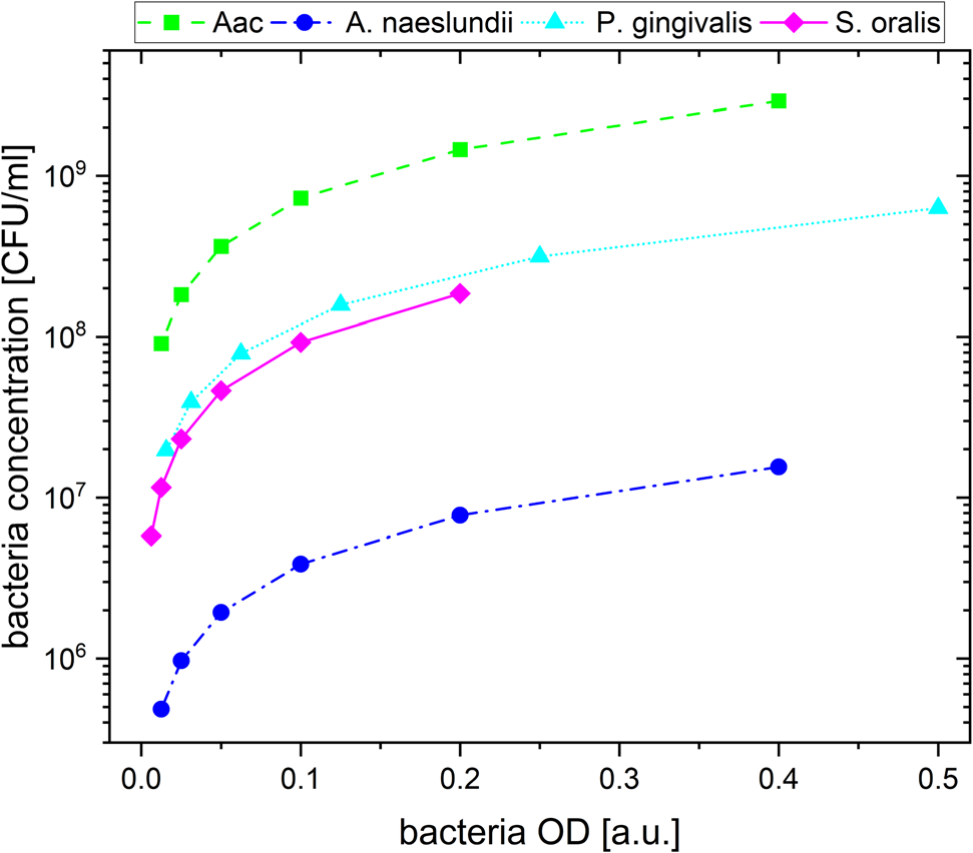
The calculated bacteria concentration as a function of bacteria OD.

For the plasmonic sensing of bacteria, samples were placed in transparent 96 well plates. First, the AuNPs were prepared at an optical density (OD) of 1 at their LSPR peak followed by a 2-fold dilution with MilliQ water, leading to 3 different AuNP dilutions at OD_AuNP_ 1, 0.5 and 0.25. The precultured bacteria were washed with autoclaved distilled water and prepared at the optical density measured at 660 nm shown in Table 2, then a 2-fold serial dilution was prepared leading to 8 bacteria dilutions for each species. These bacteria ODs were chosen based on preliminary experiments which showed distinct differences between the four oral bacteria leading to a strong response with AuNPs (OD_AuNP_ 1). To calculate the corresponding number of colony forming units (CFU), bacteria were grown as described above, adjusted to the OD as shown in Table 2 and 10-fold serially diluted in phosphate buffered saline (Biochrome GmbH, Berlin, Germany) or BHI (in case of *S. oralis*). From each dilution, 100 μL were plated on Fastidious Anaerobe Agar (Oxoid Limited) plates containing 10% sheep blood (Thermo Fisher Scientific, Waltham, Massachusetts, USA). Bacteria plates were incubated for 48 h (*S. oralis*, *A. naeslundii*, *Aac*) or 7 days (*P. gingivalis*) under the conditions described in Table 1. Afterwards, colonies were manually counted. Using the standard curve function in GraphPad Prism (v10.1.1, GraphPad Software, Boston, MA, USA), the corresponding colony forming units (CFU) were calculated for each bacterial OD. Fig. 1 shows the resulting bacteria concentration in CFU per mL as a function of bacteria OD. For sensing experiments, each well plate was prepared three times. In every well, 100 μL of the appropriate AuNP dilution were added and pictures were taken. Afterwards, 50 μL of the appropriate bacteria dilution were added. Whereas for the reference, instead of bacteria, 50 μL of autoclaved distilled water were added. Directly after the addition of bacteria, another picture was taken, after which pictures were taken after 5 min, 10 min, 20 min, 60 min and 90 min. Subsequently, the well plate was measured in the UV-vis spectrometer.

**Table 2:** The OD of bacteria used measured at 660 nm and their corresponding concentration

Bacteria	OD (a.u.)	Concentration (CFU mL^−1^)
*Aac*	0.4	2.91 × 10^9^
*A. naeslundii*	0.4	1.55 × 10^7^
*P. gingivalis*	0.5	6.3 × 10^8^
*S. oralis*	0.2	1.85 × 10^8^

### 2.4 Colorimetric analysis

Pictures of the wells were captured from above with an illuminated background using a mini LED light pad (Jusony) covered with diffusive paper for an even light distribution. All images were taken at a fixed distance from the camera. Manual acquisition was chosen with the following camera settings: f/8.0 aperture, 1/80 s exposure time, ISO: 250 without any additional exposure correction or automatic white balancing. Raw image files were used for *RGB* colour analysis. A 20 × 20-pixel array per well was selected and the average *RGB* values were extracted using MATLAB (The MathWorks, Natick, Massachusetts, USA). To evaluate the changes in colour of the individual wells after addition of bacteria, the mean *RGB* components of the pictures were extracted from each well. The difference between the green (*G*) and red (*R*) channel (*G* − *R*) was calculated using the expression, *G* − *R* = (*G* − *R*)/255 × 100%. This value highlights the colour changes from each well. Differences of blue (*B*) and red channel, *B* − *R* values were also analysed for all data sets. *B* − *R* did not yield additional information and were less sensitive to changes in the colour of the solutions after addition of bacteria.

### 2.5 Transmission electron microscopy

Bacteria at OD specified in Table 2 were incubated with AuNP (OD_AuNP_ 1) in a ratio of 2 to 1 for 90 min and centrifuged to obtain the AuNP-bacteria pellet. Pellets were prefixed overnight in 0.15 M HEPES (pH 7.35) containing 0.15 M glutaraldehyde and 0.15 M formaldehyde and then immobilized in 2% agar and cut to cubes of 2 mm size. After post fixation in 1% osmiumtetroxide (2 h) and afterwards in 1% uranyl acetate (overnight) with intermediate washing steps, the cubes were dehydrated in acetone and embedded in epoxy resin (Agar 100 resin, Agar Scientific). Ultrathin sections of 60 nm thickness were post stained with uranyl acetate and led citrate.^36^ Images were recorded in a Morgagni TEM (FEI, Eindhoven), operated in the bright field mode at 80 kV. For images of pure nanoparticles, synthesized particles at OD_AuNP_ 1 were adhered to carbon film and imaged without staining. The mean diameter was measured using ImageJ and represented as mean ± standard deviation.

## 3. Results and discussion

### 3.1 AuNP characterization

**Fig. 2:**
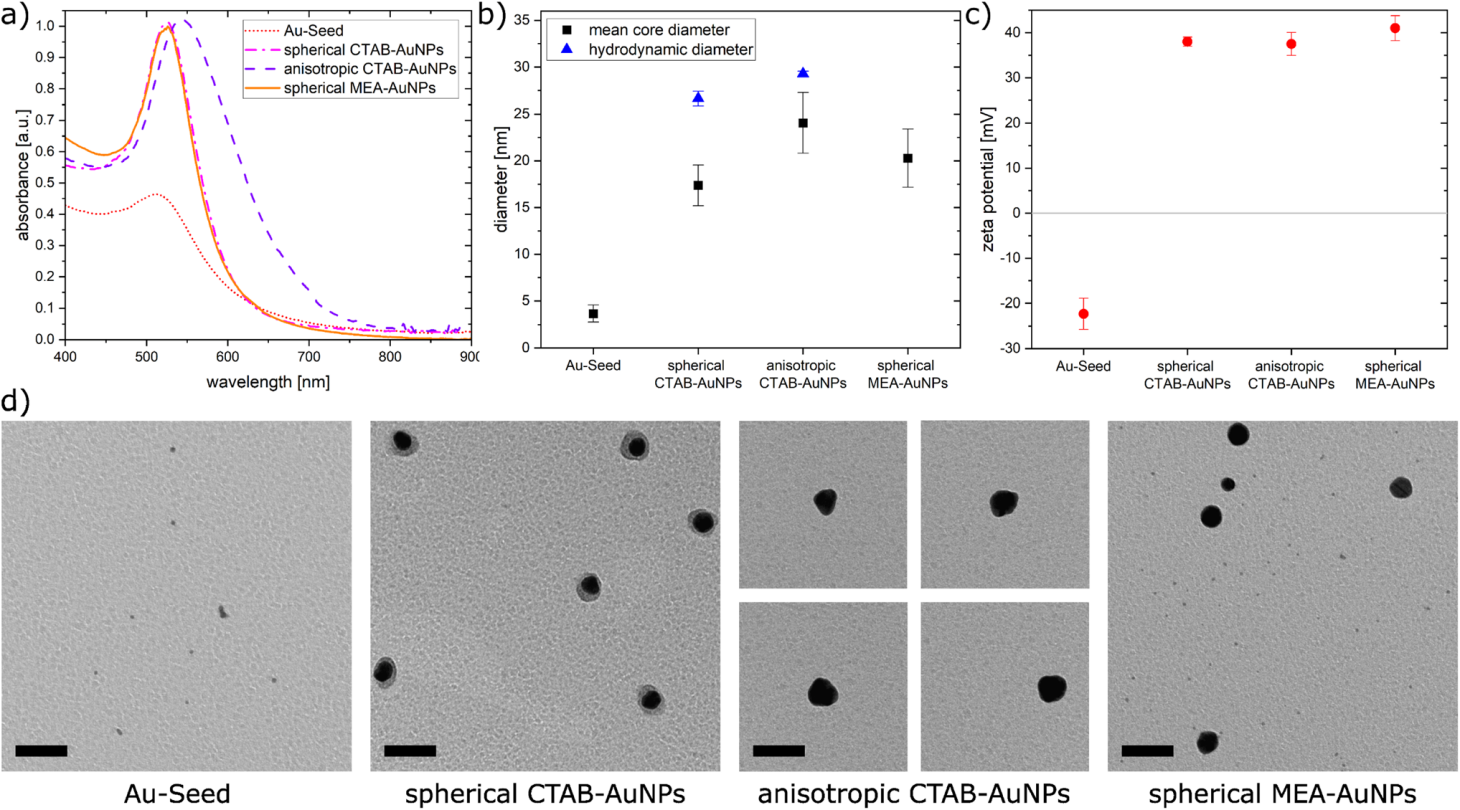
(a) The absorbance spectra of the synthesized nanoparticles (b) calculated mean core diameter from the TEM images and hydrodynamic diameter measured by dynamic light scattering, (c) plot of the zeta potential for the synthesized nanoparticles, and (d) TEM images of synthesized gold nanoparticles. Scale bar is 50 nm.

In this work, AuNPs functionalized with CTAB and MEA were synthesized to achieve a positive charge on the AuNP surfaces to allow an electrostatic interaction between the negatively charged bacteria surface and positively charged AuNP surface. Both CTAB and MEA act as stabilizers for the AuNPs in solution and provide a positive surface charge.^37,38^ Depending on their size and shape, AuNPs have a LSPR peak at a different spectral position. Fig. 2a shows the measured absorbance spectrum for the synthesized particles as well as the Au-seed (red dotted line) used for the synthesis of the CTAB-AuNPs with a LSPR peak at 512 nm. The spherical CTAB-AuNPs (pink line) and MEA-AuNPs (orange line) show a LSPR peak at 525 nm which indicates that these AuNPs have similar radii. The anisotropic CTAB-AuNPs (purple line) have a broader LSPR peak at 544 nm indicating a larger radius. Particles remained stable for more than 6 months after synthesis.

Transmission electron microscopy (TEM) was used to visualize the shape and quantitatively measure the diameter of the synthesized particles. Fig. 2b shows the hydrodynamic diameter measured by dynamic light scattering (DLS) and the mean diameter measured from the TEM images of the AuNPs. The corresponding zeta potential measurements are shown in Fig. 2c. Au-seed has a particle diameter of 3.67 nm ± 0.89 nm (41 particles counted) and appears polydisperse as small spherical AuNPs, as shown in the TEM image (Fig. 2d). Because of their relatively high polydispersity index 0.609 ± 0.002, the hydrodynamic diameter couldn't be exactly measured, since the DLS measurement is not reliable for polydispersity index >0.5.^39^ Synthesized spherical CTAB-AuNPs have a hydrodynamic diameter of 26.64 nm ± 0.78 nm and mean diameter of 17.37 nm ± 2.18 nm (87 particles counted). Values for the hydrodynamic diameter are typically larger than the mean diameter since this includes the solvate shell of the particles. Fig. 2d shows the individual TEM images for the synthesized AuNPs. Most of the spherical CTAB-AuNPs are not perfectly spherical. In contrast, the anisotropic CTAB-AuNPs appear in different shapes, such as triangle, cubic and oval with a hydrodynamic diameter of 29.27 nm ± 0.29 nm and a mean core diameter of 24.04 nm ± 3.24 nm (73 particles counted), which is the minimum dimension of the particles. Meanwhile, the spherical MEA-AuNPs have a mean diameter of 20.28 nm ± 3.12 nm (63 particles counted) and appear more spherical then the spherical CTAB-AuNPs. MEA-AuNPs and the spherical CTAB-AuNPs develop a LSPR peak at the same wavelength even though the MEA-AuNPs are larger.

To quantify an approximation of the net electric charge of the particles in water, the zeta potential was measured (Fig. 2c). Au-seed has a negative zeta potential of −22.3 mV ± 3.42 mV which is caused by trisodium citrate on the particle surface.^40^ During the synthesis, the trisodium citrate on the particle surface was exchanged with CTAB and accordingly the zeta potential becomes positive for the CTAB-AuNPs, with values for the spherical AuNPs at 38 mV ± 1.03 mV and anisotropic AuNPs at 37.5 mV ± 2.56 mV. Meanwhile, the spherical MEA-AuNPs have a higher, positive zeta potential of 41 mV ± 2.77 mV. Hence, the synthesized particles can be considered stable based on the classical definition that stable functionalized AuNPs have a zeta potential > |30| mV.^39^

In summary, stable CTAB and MEA functionalized AuNPs were synthesized in different sizes and shapes with a strong positive surface charge. Compared to the synthesized AuNPs of Verma *et al.*^27^ the produced CTAB-AuNPs in this work are smaller with a LSPR peak at a shorter wavelength and less branched anisotropic CTAB-AuNPs. Meanwhile, the synthesized MEA-AuNPs have similar characteristics, *e.g.* the LSPR peak, as described by Sun *et al.*^34^

### 3.2 AuNP aggregation to bacteria surfaces characterized by zeta potential measurements and TEM

**Fig. 3:**
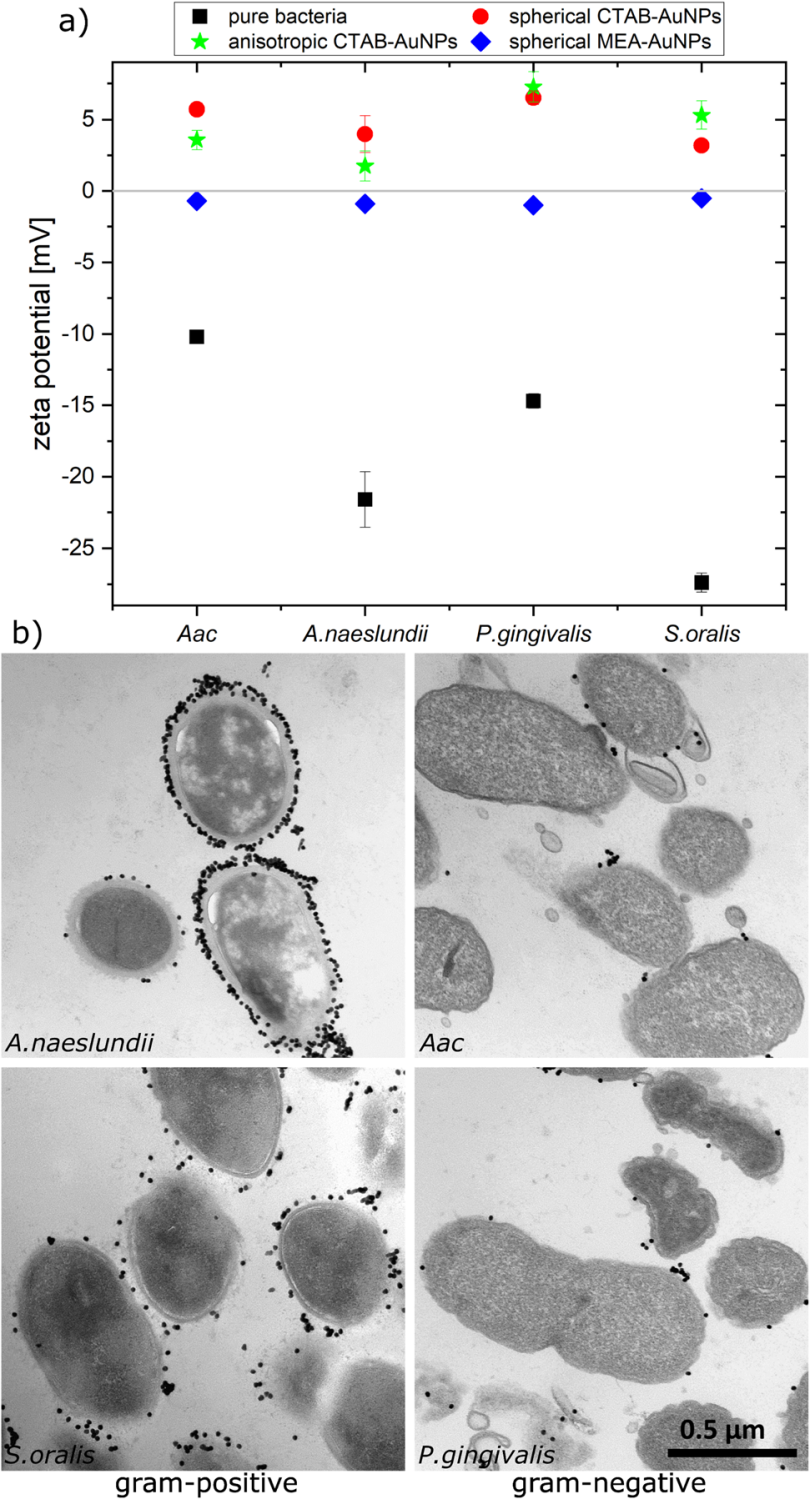
Aggregation of AuNPs on oral bacteria (*A. naeslundii*, *S. oralis*, *Aac* and *P. gingivalis*) incubated with AuNPs. (a) Plot of the measured zeta potential for different oral bacteria species. (b) TEM images showing the arrangement of spherical CTAB-AuNPs around the bacteria cell wall. Scale bar is 500 nm.

To confirm the aggregation of the synthesized AuNPs around the bacterial cell wall, the zeta potential of each bacterial strain was measured before and after addition of the different AuNPs (Fig. 3a). In water, bacteria showed a more negative zeta potential for Gram-positive bacteria (*S. oralis* and *A. naeslundii*) compared to Gram-negative bacteria (*Aac* and *P. gingivalis*). With the addition of AuNPs, the zeta potential in all bacteria increased toward ∼0 mV. These small zeta potential values depict an unstable condition and therefore aggregation of AuNPs. The increase of the zeta potential is to be expected due to the AuNPs binding on the surface of bacteria. Due to their positive zeta potential (see Fig. 2c), the AuNPs aggregate on the negatively-charged bacteria surface owing to electrostatic attraction. In comparison to CTAB functionalized AuNPs, MEA-AuNPs led to the lowest increase in zeta potential for all bacteria.

To visualize the arrangement of the AuNP aggregates on the bacteria surface, TEM images of each bacterial species were taken after the addition of spherical CTAB and MEA-AuNPs. Fig. 3b shows that the AuNPs aggregate on the bacteria surface and did not diffuse inside the bacteria. CTAB-AuNPs seemed to attach more on Gram-positive bacteria's cell walls than on Gram-negative bacteria which is consistent with the data of Verma *et al.*^28,29^ Interestingly, interaction of CTAB-AuNP to *A. naeslundii* exhibited an all-or nothing coverage, wherein either the AuNPs are arranged in a layer covering the entire bacteria or with a few isolated AuNPs attached to the bacterial surface. This could be caused by the rapid electrostatic interaction between *A. naeslundii* and AuNPs. Leading to aggregation, we assume that AuNPs interacted only to the immediate *A. naeslundii* bacteria cells they encounter. *S. oralis* showed sparse AuNP coverage with smaller aggregates. On the other hand, *Aac* and *P. gingivalis* had minimal aggregates with predominantly isolated AuNPs on their surfaces. Similar observations but with slightly varying degree of aggregation depending on the bacterial species could be seen on TEM images of MEA-AuNPs (ESI Fig. S1†).

### 3.3 Colorimetric sensing experiments

**Fig. 4:**
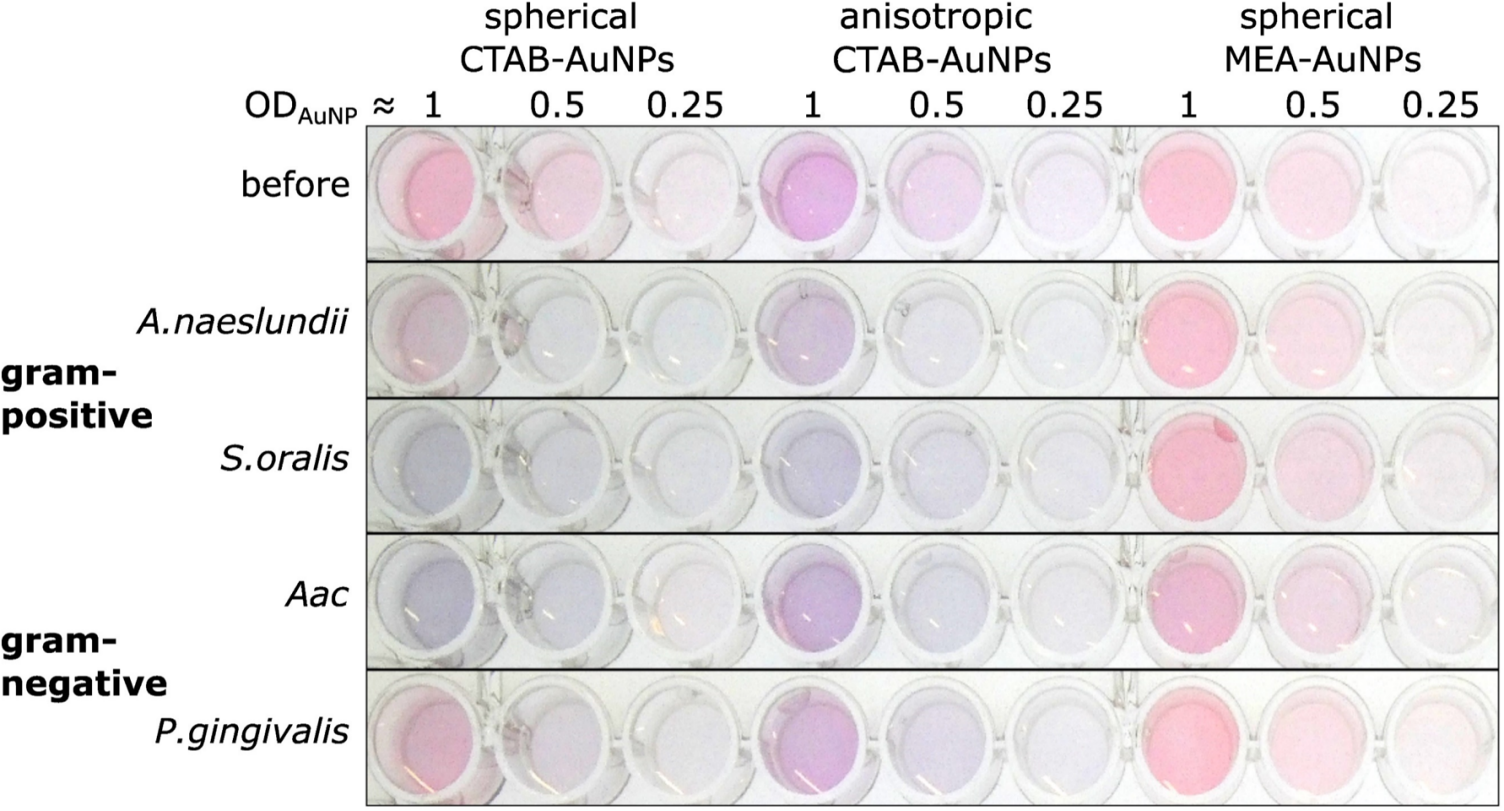
A representative image of wells containing a dispersed solution of CTAB- and MEA-AuNPs (AuNP dilution in terms of OD_AuNP_ are specified on top of the well plate) before and 10 min after the addition of oral bacteria (*A. naeslundii*, *S. oralis*, *Aac* and *P. gingivalis*) at bacteria OD listed in Table 2. Due to the differences in bacteria concentration, the relative colour changes among the species cannot be compared to each other.

The synthesized AuNPs at three different dilutions were used as an AuNP plasmonic sensor array of the oral bacteria. Fig. 4 shows a representative image of this array before and 10 min after the addition of bacteria with the ODs at corresponding concentrations listed in Table 2. A visual colour change could be observed depending on the bacterial species and AuNP dilution. For example, addition of *S. oralis* to spherical CTAB-AuNPs turned the original pink solution to bluish colour and almost colourless at OD_AuNP_ 0.25. In contrast, with the same *S. oralis* concentration to MEA-AuNPs, no visible change in colour could be observed. Addition of *Aac* to MEA-AuNPs gave a bluish tinge to the solution at OD_AuNP_ 1.

**Fig. 5:**
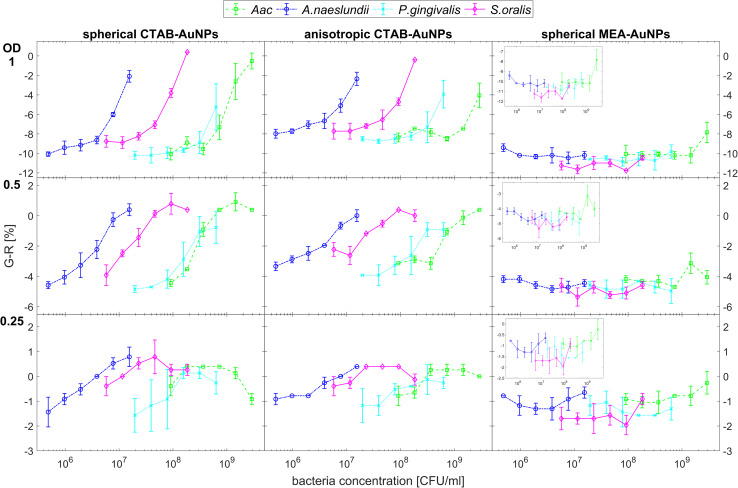
Colour change represented by average *G* − *R* values (*n* = 3) upon addition of bacteria to functionalized AuNPs. *G* − *R* were obtained using colorimetric assay of CTAB- and MEA-AuNPs 10 min after the addition of the respective oral bacteria (*A. naeslundii*, *S. oralis*, *Aac* and *P. gingivalis*). Error bars denote the standard deviation. Note that *y*-axes have different range for different AuNP dilutions to highlight the differences in *G* − *R* as a function of bacteria concentration.

Fig. 5 shows the behaviour of *G* − *R* as a function of bacteria concentration, 10 min after bacteria addition. For all species, the colour change slowly increased, with increasing bacteria concentration. Distinct changes from baseline *G* − *R* especially for CTAB-AuNPs occurred at a certain bacteria concentration threshold which is different for each species. For Gram positive bacteria, *A. naeslundii* and *S. oralis*, bacteria concentration threshold occurred at lower bacteria concentrations (<10^7^ CFU mL^−1^) compared to *Aac* and *P. gingivalis* (>10^7^ CFU mL^−1^). Some bacterial species developed a peak in *G* − *R* at a certain bacteria concentration. For example, for *S. oralis* mixed with CTAB-AuNPs, *G* − *R* values showed peaks at higher AuNP dilutions (see Fig. 5). For bacterial species mixed with MEA-AuNPs, *G* − *R* value change as a function of bacteria concentration occurred more evidently at the highest AuNP dilution (OD_AuNP_ 0.25).

**Fig. 6:**
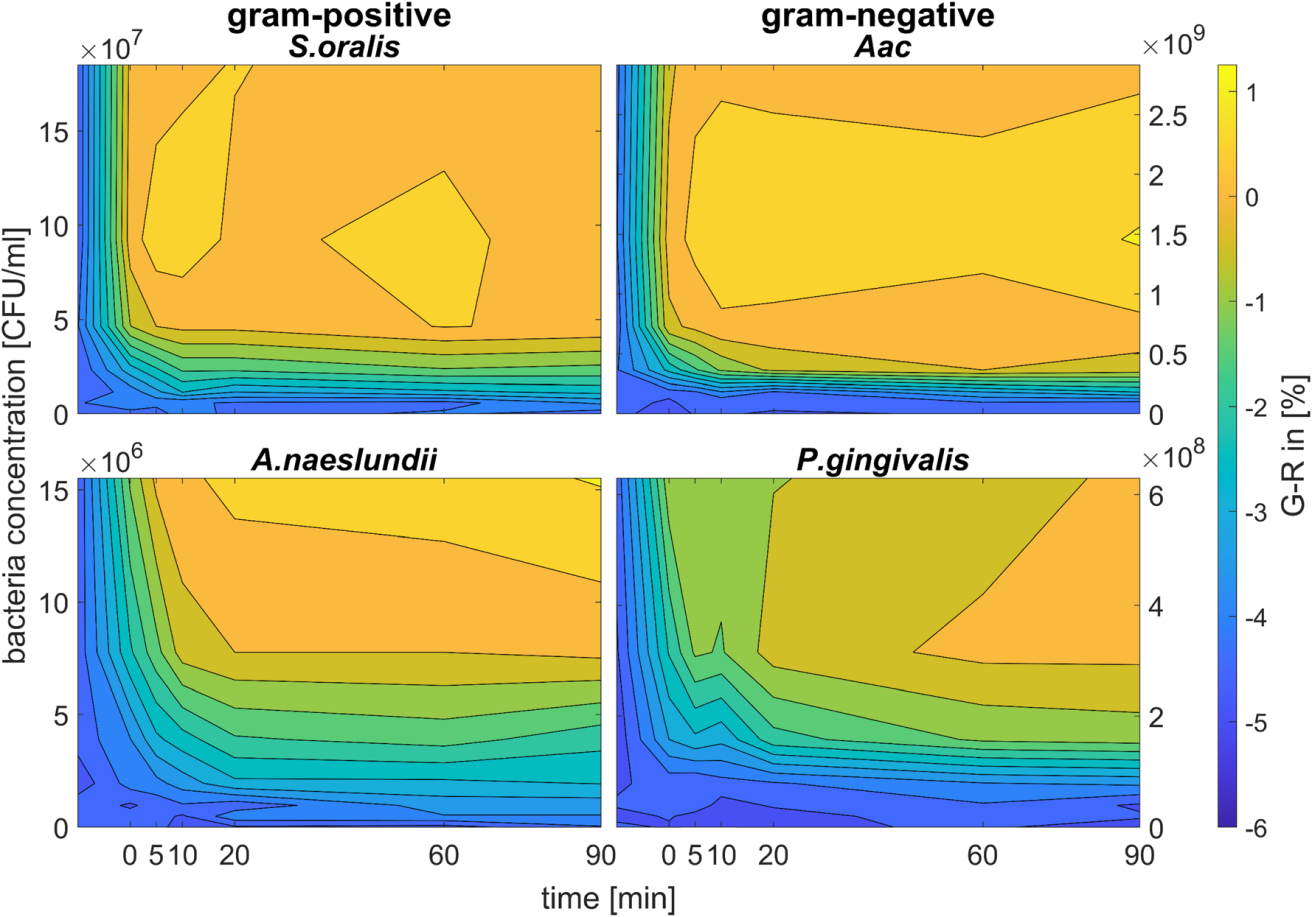
Colorimetric sensing of oral bacteria species (*A. naeslundii*, *S. oralis*, *Aac* and *P. gingivalis*). *G* − *R* values of spherical CTAB-AuNP at OD_AuNP_ 0.5 plotted as a function of incubation time.

Fig. 6 shows the colour change of the spherical CTAB-AuNPs at AuNP dilution, OD_AuNP_ 0.5, as a function of bacteria concentration and incubation time. Smaller, coccoid-shaped bacterial species, namely *Aac* and *S. oralis* showed the fastest response with most of the changes in *G* − *R* occurring in the first 10 min. On the other hand, rod-shaped *A. naeslundii* and *P. gingivalis* needed around 20 min to show their highest response. For higher AuNP dilutions, the reaction time decreased as seen in ESI Fig. S2.† When using MEA-AuNPs, not all bacteria led to a significant change in *G* − *R* (see Fig. 5 and ESI Fig. S2†). For OD_AuNP_ 0.5, the highest response was induced when adding *Aac* to MEA-AuNPs.

### 3.4 UV-vis spectroscopy

**Fig. 7:**
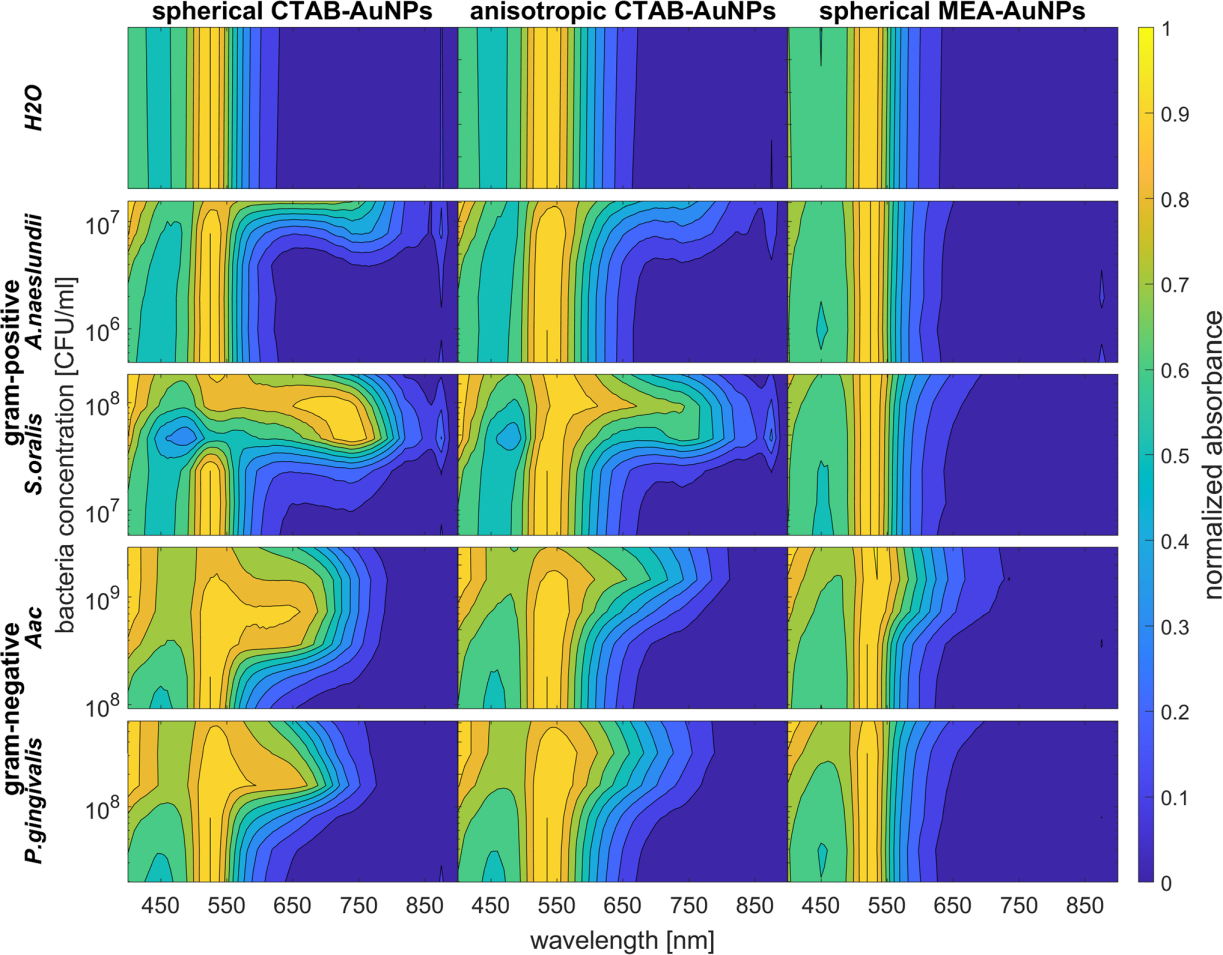
Normalized absorbance values obtained *via* UV-vis spectroscopy of CTAB- and MEA-AuNPs at OD_AuNP_ 0.5 measured 90 min after the addition of oral bacteria species (*A. naeslundii*, *S. oralis*, *Aac* and *P. gingivalis*) and water as a reference.

To confirm the results obtained using the colorimetric approach, the UV-vis absorbance spectra of the well plates were also measured after 90 min. The absorbance at OD_AuNP_ 0.5 was normalized and then plotted as a function of bacteria concentration and wavelength as shown in Fig. 7. ESI Fig. S3† shows the absorbance for other AuNP dilutions. Depending on the AuNP shape and functionalization, the changes in the LSPR position differ. The degree of AuNP aggregation around the bacteria could induce an appearance of a second absorbance peak at longer wavelengths, broaden the main absorption peak or shift the peak to longer wavelengths. For example, the aggregation of AuNPs on the surface of *S. oralis* led to a second peak at around 740 nm in spherical CTAB-AuNPs and broadened the peak of anisotropic CTAB-AuNPs up to 740 nm. Meanwhile, broadening of the absorption peak could be seen for all bacterial species interacting with MEA-AuNPs. Also, distinct differences between the bacterial species could be seen directly in the UV-vis absorbance spectra. In contrast to *S. oralis*, *Aac* spectra were broadened for all synthesized AuNPs with the strongest LSPR peak broadening, to a maximum of 650 nm, for spherical CTAB-AuNPs. Anisotropic CTAB-AuNPs were more optimal to detect *S. oralis*, while *Aac* showed low responses for both anisotropic CTAB-AuNPs and MEA-AuNPs. Consistent with the *G* − *R* analysis, MEA-AuNPs showed the strongest response toward *Aac*, corresponding to a significant change in absorbance.

**Fig. 8:**
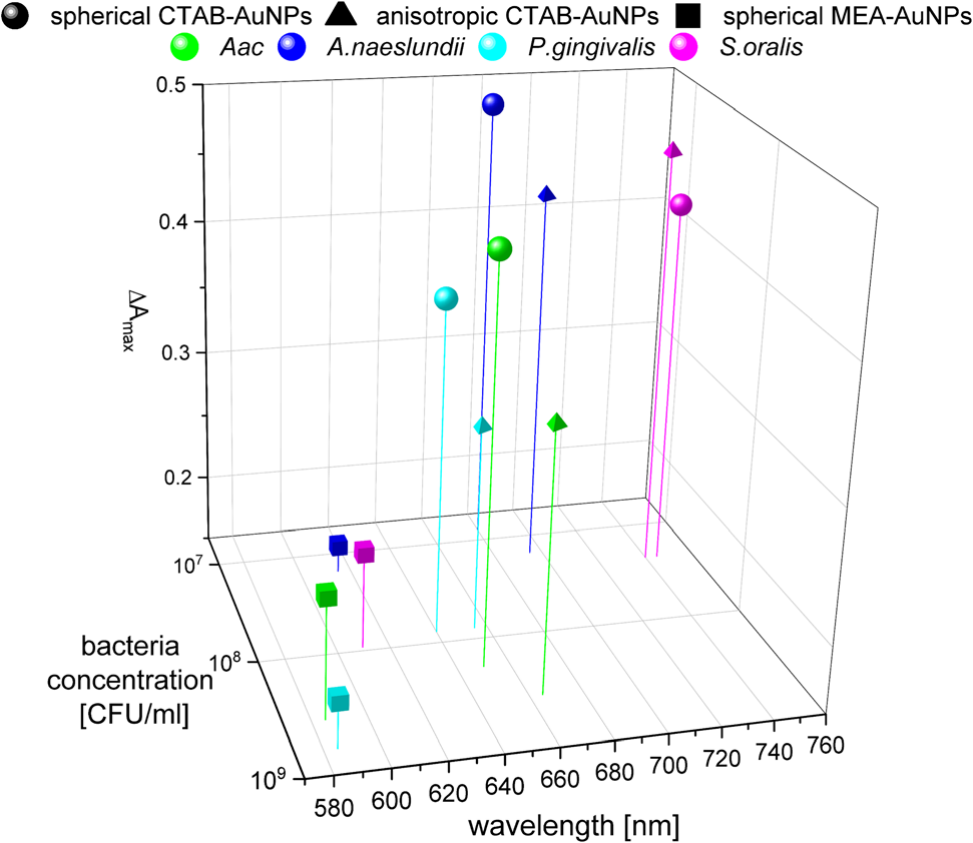
Maximum absorbance change, Δ*A*_max_ derived from UV-vis spectrometry of AuNPs at OD 0.25 and bacteria concentration saturation point, 90 min after the addition of oral bacteria.

In colorimetric assays using *RGB* components, *R* is associated with the wavelength range of 650 to 780 nm and *G* with the range of 500 to 560 nm.^41^ Thus, for better comparison of the absorbance spectra with the extracted *RGB* values, an absorbance ratio was calculated from the absorbance spectra using the equation *A*_ratio_ = *A*_*λ*_/*A*_LSPR_, wherein *A*_*λ*_ is the absorbance value at each wavelength and *A*_LSPR_ is the absorbance at the LSPR peak specifically, at 525 nm and 545 nm for the spherical AuNPs and anisotropic CTAB-AuNPs, respectively. To represent *G* − *R*, *A*_ratio_ can be calculated using *A*_*λ*_ at a wavelength within the *R* value range, for example at 700 nm (ESI Fig. S4†). Similar to *G* − *R*, the absorbance ratio did not exhibit distinct changes for MEA-AuNPs. Using the relation Δ*A* = *A*_ratio_(bacteria) − *A*_ratio_(H_2_O), the absorbance change Δ*A* at each wavelength was calculated by subtracting the *A*_ratio_ of AuNPs in water from the *A*_ratio_ of AuNPs with bacteria. For further evaluation, the maximum absorbance change Δ*A*_max_ at wavelength ≥ LSPR position were extracted. This calculation was done for AuNPs at dilution OD_AUNP_ 0.25 and the bacteria concentration saturation point (bacteria concentration where the *G* − *R* is at maximum), 90 min after bacteria addition. Fig. 8 shows the maximum absorbance change Δ*A*_max_ plotted with its corresponding wavelength and bacteria concentration saturation point. Overall, for CTAB-AuNPs, Δ*A*_max_ occurred at longer wavelength compared to MEA-AuNPs. Furthermore, Δ*A*_max_ increased with wavelength. This indicated that a higher plasmonic response corresponds to an UV-vis absorbance shift to higher wavelengths. In general, Δ*A*_max_ appeared for all bacteria and functionalized AuNPs in the range between 580 to 750 nm. A relationship could be seen between the Δ*A*_max_ value, its wavelength and the bacterial species. Gram-negative bacteria (*Aac* and *P. gingivalis*) showed lower Δ*A*_max_ at shorter wavelengths compared to the Gram-positive bacteria (*A. naeslundii* and *S. oralis*). To compare the *G* − *R* data with the UV-vis data, the absorbance ratio was further calculated at 625 nm (*A*_625_ = *A*_625nm_/*A*_LSPR_) to accommodate the absorbance change induced by the aggregation of MEA-AuNPs (ESI Fig. S5†). *A*_625_ was able to depict responses for *Aac* as well as *P. gingivalis* for all synthesized AuNPs.

### 3.5 Discussion

Our work demonstrates that oral bacterial species show a unique set of responses using our synthesized AuNP biosensor array. CTAB and MEA were used to functionalize AuNPs. Addition of bacteria to the functionalized AuNPs induced the aggregation of AuNPs around the bacterial membrane due to electrostatic interactions and consequently, plasmonic coupling by Coulomb interactions^42^ resulting in a visible colour change in solution. Colorimetric sensing using *RGB* values (*G* − *R*) could reliably determine the degree of aggregation of AuNPs on the bacteria. The concentration of bacteria where a *G* − *R* peak occurs can be interpreted as the ratio of bacteria to AuNP concentration at which most of the AuNPs aggregate on the bacterial membrane. Increasing the bacteria concentration beyond this saturation point reduces the amount of AuNP aggregates per bacteria, therefore reducing the *G* − *R* value. As an analytical method to detect each bacterial species, *G* − *R* already provides accurate results with high sensitivity, especially for low AuNP dilutions. It even further increases in sensitivity with higher AuNP dilutions. However, a compromise between sensitivity and accuracy must be also considered. With increasing AuNP dilution, *G* − *R* provide less precise results with higher standard deviations due to decreased *G* − *R* values.

Among the functionalized AuNP tested, CTAB-AuNPs exhibit *G* − *R* values that provide a reliable indication of aggregation. In contrast, for MEA-AuNPs, *G* − *R* is less sensitive and therefore less suitable to observe the aggregation at the dilutions tested. Since MEA-AuNPs aggregation with bacteria showed most of their absorbance changes outside the range of detectable colour changes using *G* − *R* (<650 nm), these changes cannot be fully represented by *G* − *R* values, despite that MEA-AuNPs were aggregated on the bacteria as confirmed by TEM imaging (see ESI Fig. S1†). Analysis using the absorbance ratio *A*_625_ from UV-vis spectroscopy provided a more accurate approach in detecting the MEA-AuNPs aggregation particularly for the highest AuNP dilution.

The sensitivity of the biosensor array, in terms of bacteria and AuNP concentration, also highly depends on the AuNPs shape and size. The highest sensitivity was obtained using spherical CTAB-AuNPs, followed by anisotropic CTAB-AuNPs and lastly, MEA-AuNPs. Interestingly, this observation differs from the results of Verma *et al.*^29^ who observed a higher sensitivity for anisotropic CTAB-AuNPs than for spherical particles. This difference might be caused by the smaller and less branched CTAB-AuNPs used in our work. Depending on the bacterial species and their OD, the sensitivity varies in the bacteria OD range of 0.00625 to 0.025 with the best sensitivity according to bacteria OD following the order, *S. oralis* > *Aac* > *A. naeslundii* > *P*. *gingivalis*. Considering the bacteria concentration, the sensitivity range lies between 10^5^ to 10^7^ CFU mL^−1^ with the best sensitivity following the order, *A. naeslundii* (4.85 × 10^5^ CFU mL^−1^) > *S. oralis* (5.78 × 10^6^ CFU mL^−1^) > *P. gingivalis* (3.94 × 10^7^ CFU mL^−1^) > *Aac* (9.1 × 10^7^ CFU mL^−1^).

The specificity in plasmonic response depending on the bacterial species can be attributed to the species-dependent variation in cell envelope characteristics. Gram-positive bacteria have a cell envelope containing lipoteichoic and wall teichoic acids which contribute a negative charge *via* their phosphoryl and carboxylate groups. For Gram-negative bacteria, the thinner cell envelope contains a few peptidoglycan layers with an additional membrane containing lipid A and lipopolysaccharides (LPS).^43–46^ As shown in our zeta potential measurements and confirmed by others,^44,45^ Gram-negative bacteria have lower (more positive) negative surface charge compared to Gram-positive bacteria leading to smaller LSPR shifts. From our TEM images, the AuNP aggregates attached to Gram-negative bacteria are predominantly isolated and smaller-sized, leading to lower plasmonic responses. This is also confirmed by our UV-vis analysis, showing that changes in maximum absorption (Δ*A*_max_) appear at shorter wavelengths for *Aac* and *P. gingivalis*, both Gram-negative bacteria. As expected, *A. naeslundii* and *S. oralis* showed higher plasmonic responses, with their higher negative surface charge.

In general, for Gram-positive bacteria, AuNPs are expected to be distributed on a larger bacteria surface area, forming a net of big aggregates that cover the entire bacteria surface, leading to a higher plasmonic response compared to Gram-negative bacteria. However, we also observed differences with respect to AuNP coverage within the same Gram-stained group. TEM images showed that isolated but almost periodic small AuNP aggregates are attached on *S. oralis*. In contrast, layers of AuNPs with small interparticle spacings were observed for *A. naeslundii*. Adhesive fimbriae or surface fibrils have been observed in both early colonizers, *S. oralis* and *A. naeslundii* as well as *P. gingivalis*,^47^ which play an important role in bacterial adhesion. *S. oralis* has been shown to exhibit varying distribution of both long and short fimbriae depending on the subspecies^48^ which can be architecturally and genetically different from *A. naeslundii* fimbriae.^49^ MEA-AuNP seemed to localize more on *P. gingivalis* fimbriae (ESI Fig. S1†) as we observed AuNPs not directly attached to the membrane but located in the vicinity of the cells. *Aac* loses its fimbriated phenotype and adopts a non-fimbriated smooth-colony in an *in vitro* culture.^50^ In contrast to *Aac*, the LPS of *P. gingivalis* lacks heptose and 2-keto-3-deoxyoctonate.^51^ Additional to the membrane envelope structure, the size and shape of the bacterial species seemed to play a role in their plasmonic response, especially their evolution over time. The smaller coccoid-shaped bacterial species (*S. oralis* and *Aac*) reacted faster to the AuNPs compared to the larger rod-shaped bacterial species (*A. naeslundii* and *P. gingivalis*) as seen in Fig. 6.

Depending on the functionalization and shape, AuNPs showed a distinct plasmonic response. The cationic surfactant CTAB forms a micelle or bilayer structure around the AuNPs^52^ and electrostatic binding occurs with its ionic head. The SH group of MEA binds to gold *via* a strong sulphur–metal bond^53^ and the positively charged amino groups are free to electrostatically interact with the negatively charged bacteria. Since we observed very similar AuNP-bacteria binding configuration regardless of the functionalization used (Fig. 3b and ESI Fig. S1†), AuNP aggregation on each bacterial species seemed to depend more on the bacteria membrane properties rather than the functionalization agent. But the corresponding plasmonic response of CTAB and MEA AuNPs interacting with bacteria highly differs. CTAB-AuNPs exhibited higher plasmonic response (based on *G* − *R* value or LSPR shift) compared to MEA-AuNPs when aggregated to bacteria. Higher responses were also observed when CTAB-AuNP aggregate on Gram-positive bacteria such as *A. naeslundii* and *S. oralis* compared to Gram-negative bacteria. Interaction of MEA-AuNPs to bacteria induced mainly a broadening and a minimal shift of the LSPR peak. We attribute this partially to the more spherical shape of MEA-AuNPs compared to CTAB-AuNPs but also to the less efficient aggregation of MEA-AuNPs to the bacteria. MEA-AuNPs exhibit lower effective surface area and spatial extent which can lead to a lower overall plasmonic response with bacteria.^27^ Furthermore, to induce a visible colour shift, the aggregated AuNPs must be significantly higher than the background signal from non-aggregated or dispersed MEA-AuNPs.^54^ Despite the aggregation of MEA-AuNPs to Gram-positive bacteria, this was not sufficient to overcome the background. For future studies, plasmonic response can be enhanced by synthesizing larger MEA-AuNPs that can provide a stronger plasmonic coupling and presumably a more significant colour shift.

On the other hand, as shown in the UV-vis spectra, MEA-AuNPs showed a strong response to *Aac* (see Fig. 7) which can be useful in distinguishing this particular bacterial species from other bacteria. Sun *et al.*^34^ also showed that spherical MEA-AuNPs highly interact with the LPS at the outer cell membrane of Gram-negative *E. coli* bacteria. The stronger response of MEA-AuNPs toward *Aac* but not to *P. gingivalis* could be due to differences in their LPS components as discussed earlier.^27^

Although the results showed that the technique could effectively distinguish between different bacterial species in a monospecies culture, this method still poses some limitations and considerations must be made before using the AuNP biosensor array in a point-of-care setting. Plasmonic responses can be affected by the presence of contaminations such as salt or metal ions which can induce unwanted aggregation of AuNPs. Especially in oral cavity, where bacteria would be mixed with saliva, nonspecific AuNP aggregation could be a major concern.^55^ The specificity of the electrostatic interaction between the positively charged nanoparticles and the negatively charged bacteria can be improved by resuspending the AuNP with a stabilizer as shown previously.^28,29,56^ Prior to use, the AuNP biosensor array would need to be trained for each bacterial species of interest. This could still be a challenging prospect in a complex microbiome setting such as the oral activity. However, it has already been shown that the functionalized AuNP detection method presented in this work can distinguish between polymicrobial samples.^56^ The simplicity of this colorimetric method without the need for highly technical equipment or extensive training are advantages over conventional methods especially in the context point-of-care diagnostics. Future prospects include application of this technique in dental medicine by detecting presence of pathogenic oral bacteria in biofilms, *e.g.*, in dental implants to ensure a timely intervention.

## 4. Conclusion

We developed an easy to use and fast sensor composed of spherical and anisotropic CTAB-AuNPs and spherical MEA-AuNPs to detect different oral bacterial species using colorimetric assay and UV-vis spectroscopy. We showed that this AuNPs biosensor array could distinguish among the oral bacterial species based on their plasmonic response. The colorimetric responses depended on a rich and complex interplay between the AuNP concentration, shape, size and functionalization, as well as the bacterial species, their membrane features and concentration. Overall, the colorimetric assay was suitable to provide information on the unique set of responses for each bacterial species with sufficient sensitivity. Therefore, this technique is promising to provide a fast and simple method for oral bacteria identification for point-of-care diagnosis.

## Author contributions

MLT-M and AH conceived the presented study and supervised the project. CW performed the experiments and formal analyses. DL developed Matlab scripts used for *RGB* analysis. MH provided critical inputs in the nanoparticle synthesis and characterization. JH performed transmission electron microscopy and interpreted the images of all samples. KD-N provided support and technical know-how in bacteria preparation and cell counting. AH, KD-N and MS acquired funding. CW and MLT-M wrote the original draft. All authors provided critical feedback and approved the final version of the manuscript.

## Conflicts of interest

There are no conflicts to declare.

## Supplementary Material

NA-006-D3NA00477E-s001
