# Supplementary material for: Colorimetric detection of oral bacteria using functionalized gold nanoparticles as a plasmonic biosensor array
Source: Nanoscale Adv. 2024 Feb 8;6(5):1447–59. doi: 10.1039/d3na00477e (PMC10898432; doi:10.1039/d3na00477e)
Supplement: NA-006-D3NA00477E-s001 [file NA-006-D3NA00477E-s001.pdf]

Electronic Supplementary Material (ESI) for Nanoscale Advances.
This journal is © The Royal Society of Chemistry 2024

# Supplementary Information: Colorimetric sensing of oral bacteria using a functionalized plasmonic biosensor array

Christina Wenck[a,e] Dorthe Leopoldt[a,e], Mosaieb Habib[b,e], Jan Hegermann[d], Meike Stiesch[c,e], Katharina Doll-Nikutta[c,e],

Alexander Heisterkamp[a,e], Maria Leilani Torres-Mapa[a,e]

[a]Institute of Quantum Optics, Leibniz University Hannover, [b]Institute of Inorganic Chemistry, Leibniz University Hannover, [c]Department of Prosthetic Dentistry and Biomedical Materials Science, Hannover Medical School, [d]Research Core Unit Electron Microscopy, Institute of Functional and Applied Anatomy, Hannover Medical School, [e]Lower Saxony Centre for Biomedical Engineering, Implant Research and Development (NIFE)

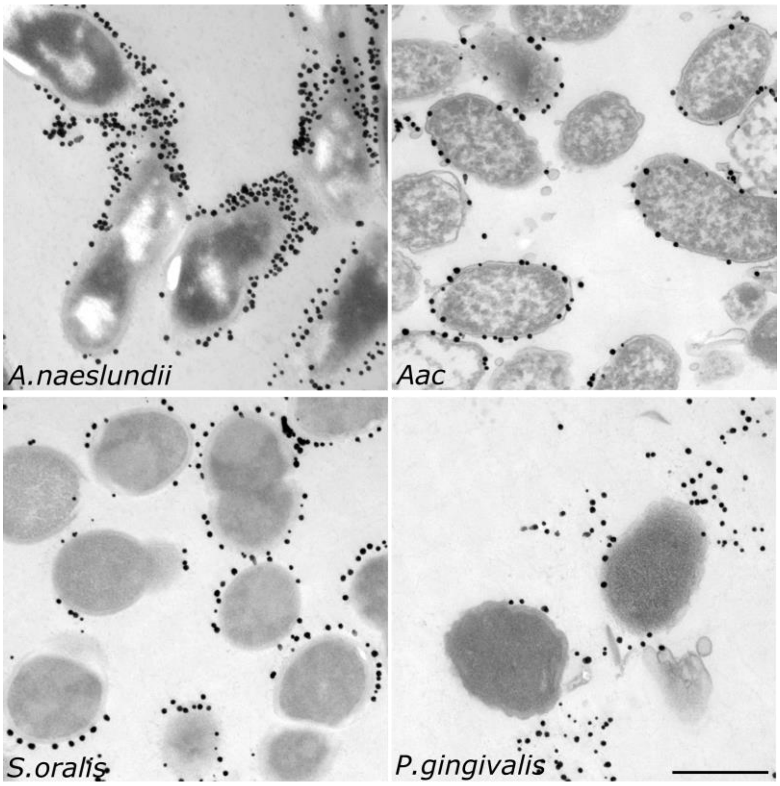


SI Figure 1: Transmission electron microscopy (TEM) of MEA-AuNP incubated with oral bacteria.

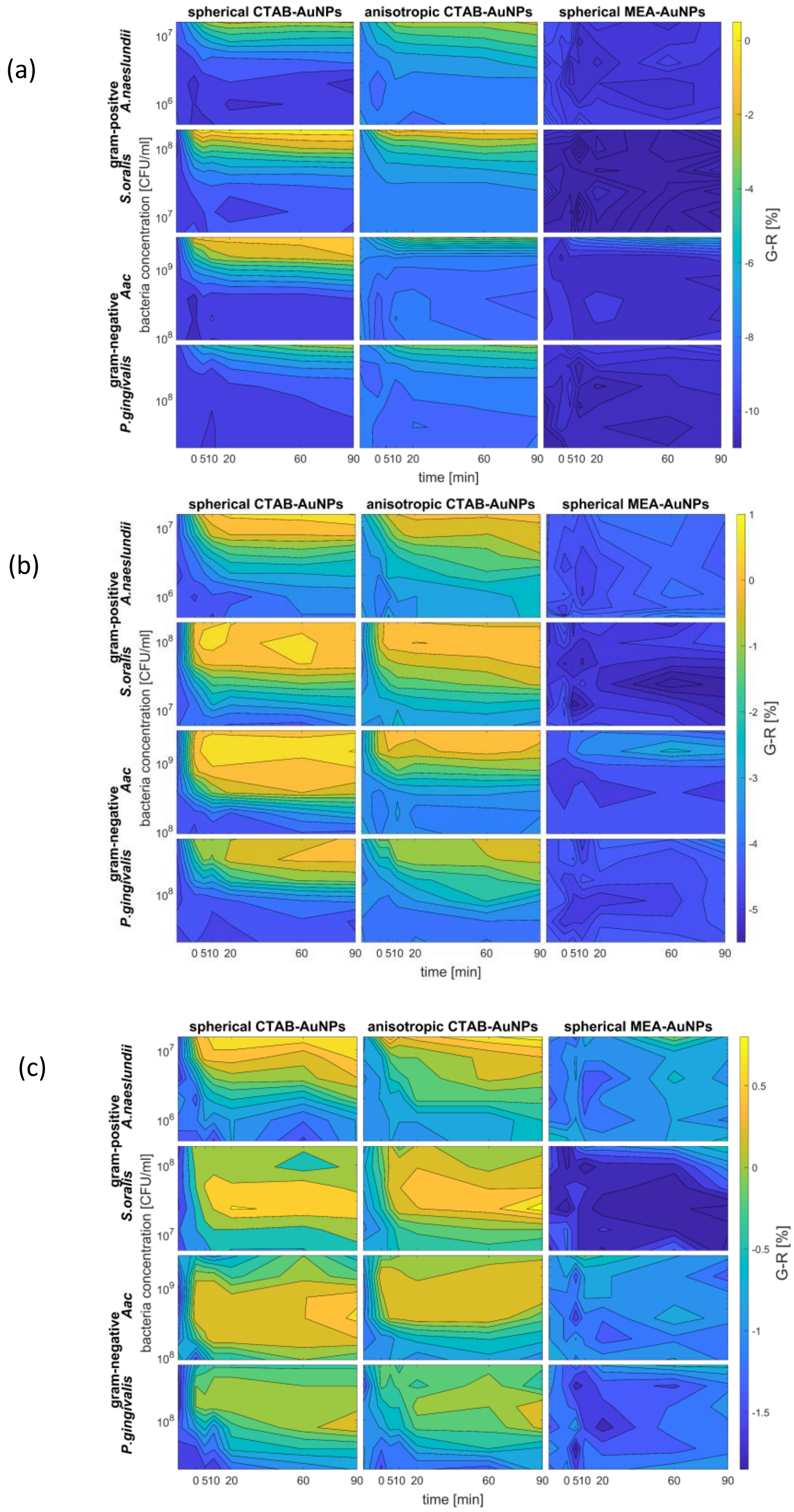


SI Figure 2: G-R values as a function of incubation time for varying AuNP dilutions (a) AuNP OD 1 (b) AuNP OD 0.5 (c) AuNP OD 0.25

(a)

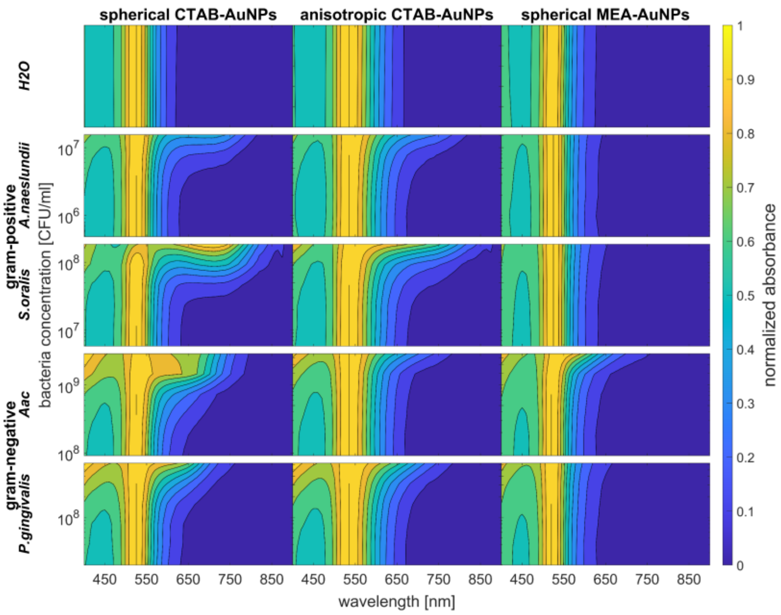


(b)

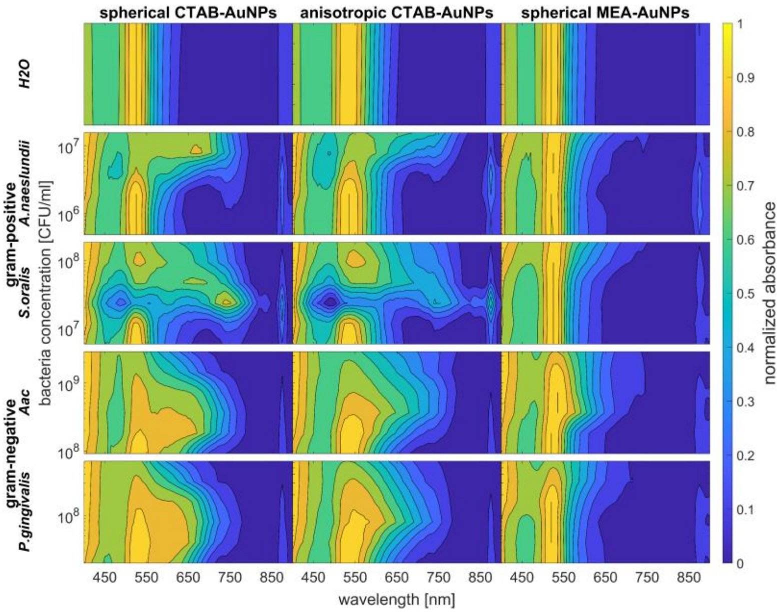


SI Figure 3: Normalized absorbance of CTAB- and MEA-AuNPs measured 90 min after the addition of oral bacteria. a) AuNP OD 1 and b) AuNP OD 0.25.

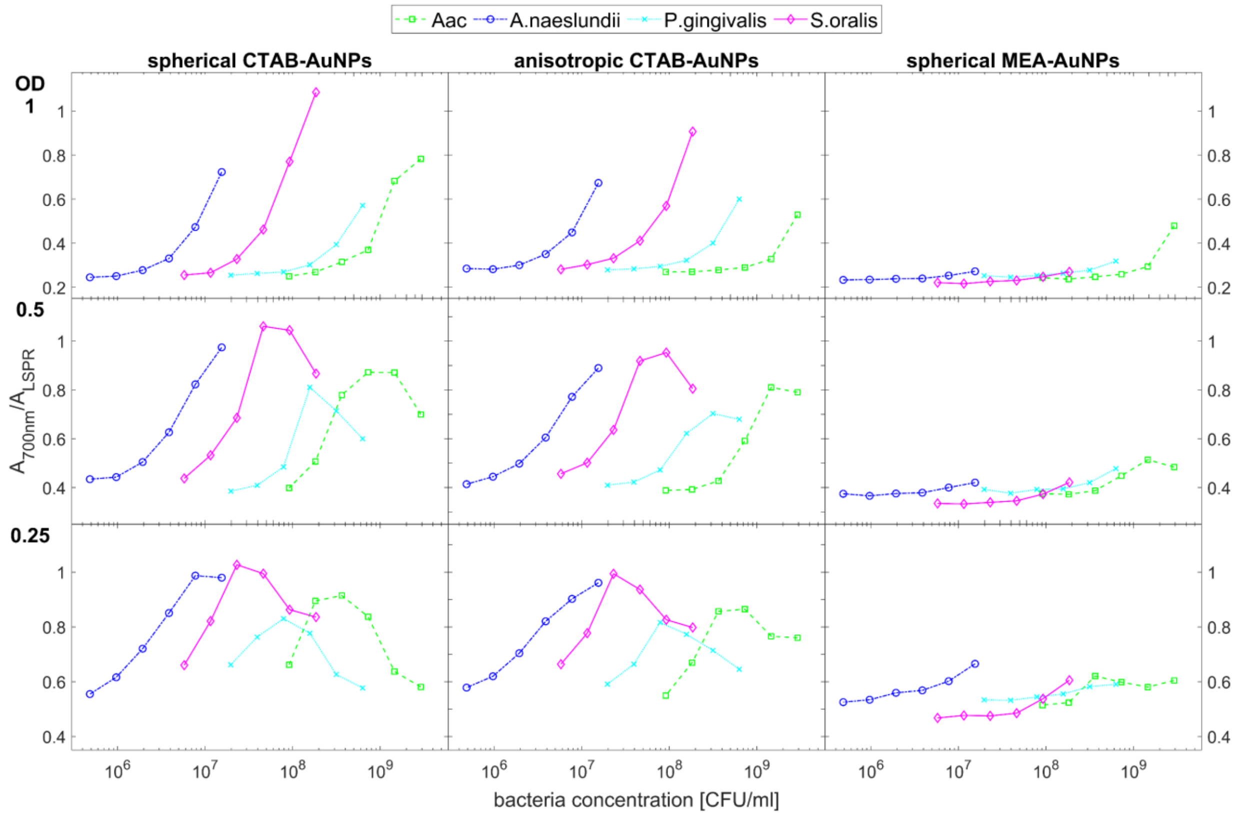


SI Figure 4: Calculated $A_{G2R}$ = $A_{700}$ values of CTAB- and MEA-AuNPs 90 min after the addition of bacteria.

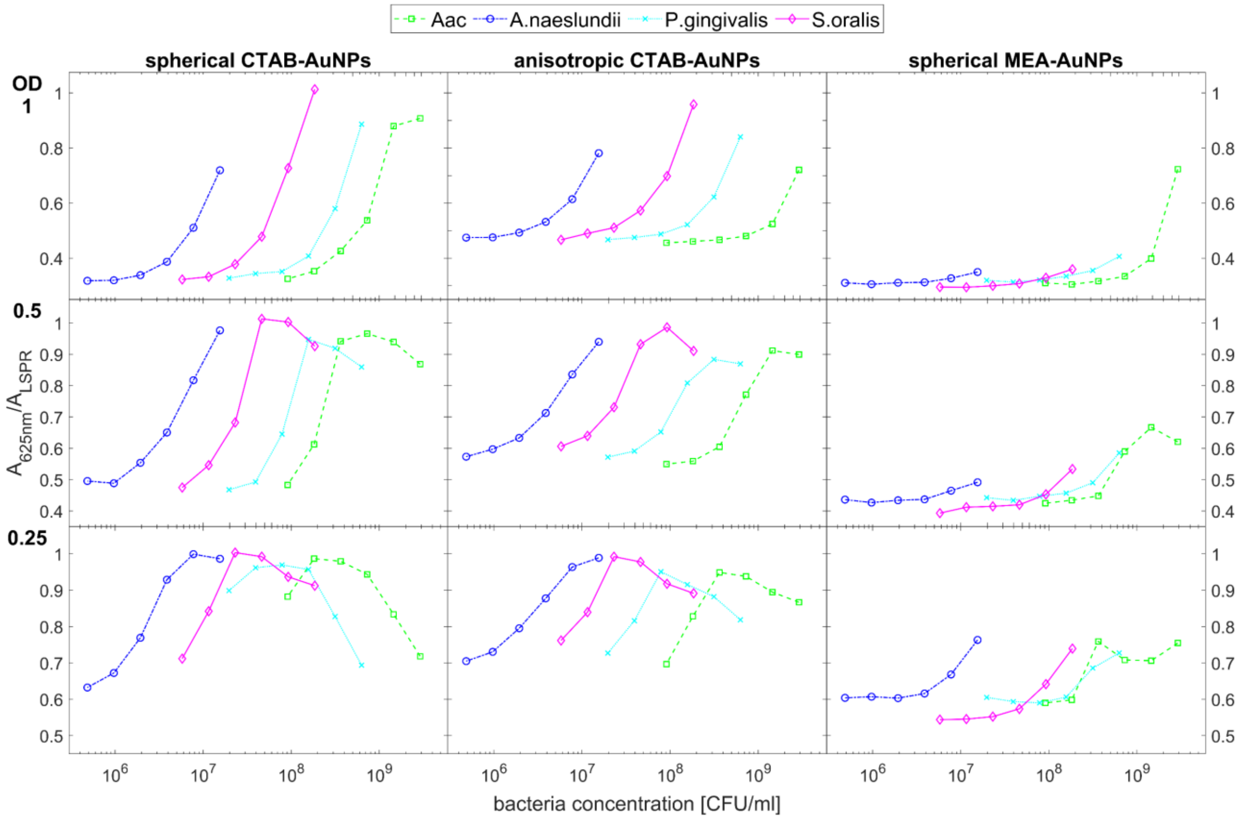


SI Figure 5: Calculated $A_{625}$ values of CTAB- and MEA-AuNPs 90 min after the addition of oral bacteria.
